# The Paradigm Shift of Using Natural Molecules Extracted from Northern Canada to Combat Malaria

**DOI:** 10.3390/idr16040041

**Published:** 2024-06-26

**Authors:** Alexandra Bourgeois, Juliana Aline Souza Lemos, Stéphanie Roucheray, Audrey Sergerie, Dave Richard

**Affiliations:** 1 Centre de Recherche en Infectiologie, CRCHU de Québec-Université Laval, Quebec City, QC G1V 4G2, Canada; alexandra.bourgeois@crchudequebec.ulaval.ca (A.B.); julianalemos@aluno.fiocruz.br (J.A.S.L.); stephanie.roucheray@crchudequebec.ulaval.ca (S.R.); audrey.sergerie@crchudequebec.ulaval.ca (A.S.); 2 Department of Microbiology-Infectious Diseases and Immunology, Faculty of Medicine, Laval University, Quebec City, QC G1V 0A6, Canada

**Keywords:** malaria, natural compounds, northern environments, northern molecules, Canadian boreal forest, metabolites, antiparasitics, antiplasmodials, Labrador tea, mortiamides

## Abstract

Parasitic diseases, such as malaria, are an immense burden to many low- and middle-income countries. In 2022, 249 million cases and 608,000 deaths were reported by the World Health Organization for malaria alone. Climate change, conflict, humanitarian crises, resource constraints and diverse biological challenges threaten progress in the elimination of malaria. Undeniably, the lack of a commercialized vaccine and the spread of drug-resistant parasites beg the need for novel approaches to treat this infectious disease. Most approaches for the development of antimalarials to date take inspiration from tropical or sub-tropical environments; however, it is necessary to expand our search. In this review, we highlight the origin of antimalarial treatments and propose new insights in the search for developing novel antiparasitic treatments. Plants and microorganisms living in harsh and cold environments, such as those found in the largely unexploited Northern Canadian boreal forest, often demonstrate interesting properties that are not found in other environments. Most prominently, the essential oil of *Rhododendron tomentosum* spp. *Subarcticum* from Nunavik and mortiamides isolated from *Mortierella* species found in Nunavut have shown promising activity against *Plasmodium falciparum*.

## 1. Introduction

Tropical diseases remain an immense burden to numerous low- and middle-income countries, most prominently parasitic diseases such as malaria, trypanosomiasis, leishmaniasis, Chagas disease, schistosomiasis, lymphatic filariasis and helminthiases [1]. Tropical diseases globally affect billions of individuals of low-income countries situated in Asia, Latin America and Sub-Saharan Africa. Despite this, only a small fraction of global funds dedicated to health are allotted annually for the study of neglected tropical diseases and the effort to alleviating their burden [2]. 

Malaria remains the heavy hitter and the burdens attached to this disease endure in many countries. In 2022, 249 million malaria cases were reported, a marked increase of 5 million cases compared with 2021 [3]. Malaria in humans is caused by six species of parasites from the genus *Plasmodium*, each having different severity and geographical distributions. *Plasmodium falciparum* is the most prominent species causing the bulk of malaria cases every year and is mostly present in sub-Saharan Africa. *P. falciparum* is also the most virulent species infecting humans, making it responsible for the majority of malaria deaths. *P. ovale wallikeri*, *P. ovale curtisii*, *P. vivax*, *P. malariae* and *P. knowlesi* are distributed in different tropical and sub-tropical regions. *P. vivax* is present in higher numbers than *P. falciparum* in South-East Asia, South America and the Western Pacific. *P. ovale* is present in tropical Africa alongside *P. falciparum*, while *P. knowlesi* infections occur within restricted forested regions of South-East Asia. *P. malariae* infections are widespread and can occur in all regions where malaria transmission can occur [4]. Mild malaria cases are accompanied by flu-like symptoms including fever, chills, headaches, muscle aches, tiredness, nausea, vomiting and diarrhea. If left untreated, more severe symptoms of malaria can appear, including kidney failure, seizures, mental confusion and coma, which could lead to death [5]. 

**Figure 1:**
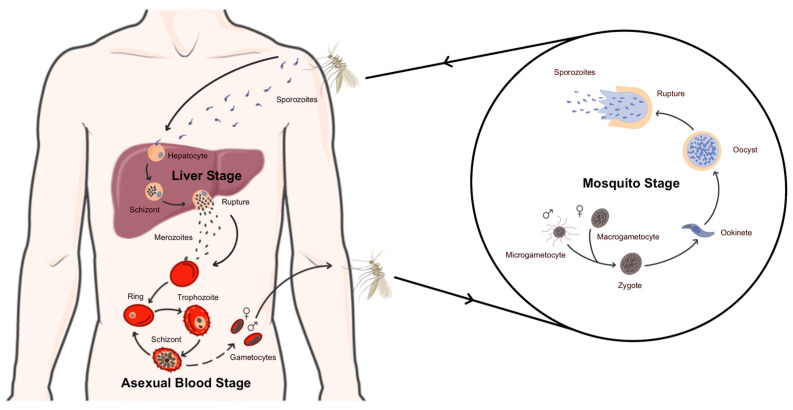
Life cycle of *Plasmodium falciparum*.

The malaria parasite has a complex life cycle necessitating the presence of two hosts, the human host and the insect vector, the *Anopheles* mosquito, as described in Figure 1. The sporozoite form of the parasite makes its entrance into the human host during an infected female *Anopheles* mosquito’s blood meal [6]. The sporozoites that were deposited in the dermis by the mosquito migrate to the liver and proceed to invade hepatocytes [7]. Once established in the hepatocytes, the parasite undergoes schizogony [8]. The mature schizont causes the cell to rupture, releasing newly formed merozoites which gain entrance into the bloodstream. This marks the beginning of the erythrocytic cycle, the stage of the disease responsible for all clinical symptoms [9]. Each cycle lasts approximately 48 h and is characterized by the progression of parasites through three distinct morphological stages named ring, trophozoite and schizont [10]. Following multiple cycles of replication, a small proportion of parasites differentiate into gametocytes, the transmittable sexual form of the parasite. Once reintroduced into the mosquito, the parasite goes through a cycle of sexual replication. As demanded by its unique biological niche, *Plasmodium* parasites possess a set of unique organelles essential for its survival. These include the apicoplast, the food vacuole and the micronemes, rhoptries and dense granules, forming the apical complex. 

A set of new risks and challenges caused by a rapidly changing world have greatly impacted the global fight against malaria. This is visible in the increase in malaria cases reported during 2022, high above the predicted numbers before the COVID-19 pandemic [3]. As described in the 2023 World Malaria Report released by the World Health Organization (WHO), climate change, conflict, humanitarian crises, resource constraints and diverse biological challenges represent an extreme risk in not only the spread but also the burden of the disease. In fact, the latest edition of the World Malaria Report is the first to dedicate a chapter on climate change and its impact on malaria. Climate change affects several different aspects of infectious diseases, especially parasitic infections. Climate and temperature are intrinsically important for the development and life habits of vectors and the growth of parasites within them. The habits of humans and animals are also often affected as well [2]. Extreme weather events such as increased rainfall and heatwaves can modify vector behavior and thus transmission. This variability can make an environment ideal for vector proliferation. Studies have shown that regions suitable for the transmission of malaria and other neglected tropical diseases will broaden with warming and an unpredictable climate, exacerbating the burden on afflicted areas with fragile healthcare infrastructure and limited resources [11].

The occurrence of malaria importation and subsequent transmission in countries where it has been eradicated has also increased as a consequence of climate change. An increased number of imported malaria cases is posing significant risk to the reestablishment of malaria in susceptible areas such as China [12], warmer regions of Europe [13] and Southern regions of North America [14,15]. Patients contracting a malaria infection for the first time could develop severe disease and overwhelm hospitals that are unprepared to treat these types of infections [16]. A prominent example occurred during 2023 with the US Centers for Disease Control and Prevention (CDC) reporting between May 18th and July 17th eight cases of locally acquired *P. vivax* malaria in Florida and Texas. Importantly, a case of locally acquired *P. falciparum* malaria was reported in Maryland in August. Of note, the last time locally acquired malaria was recorded in the United States was in 2003, and before that, the recorded cases of malaria were sporadic since the late 1950s, and this is despite the USA obtaining the certification of malaria eradication in the 1970s. It was to be expected that a potential disease resurgence could happen in Southern USA as a consequence of climate change and expanding mosquito habitats. Species of *Anopheles* mosquitoes capable of transmitting malaria if infected are well established in many states and are making their way up well into North America [17,18]. Climate variability can also cause population displacement, resulting in an influx of population without immunity within endemic areas [19]. As malaria is spreading alongside resistance to front-line drugs, it is crucial to find new alternative and cost-effective treatments for malaria [2]. 

Extensive literature reviews are available on the use of natural molecules from northern environments such as the Canadian boreal forest and from polar marine organisms for the treatment of different ailments and their symptoms [20,21]. However, a gap in the knowledge is present in regard to how these molecules could be employed to treat parasitic diseases. In this work, we first attempted to highlight the relevance of the use of these molecules and then emphasize their potential use in the treatment of parasitic diseases such as malaria.

## 2. From Natural Remedies to Synthetic Molecules and Back Again

The history of malaria is closely intertwined with that of humankind. Populations plagued by malaria adapted through the development of natural remedies to alleviate fever symptoms and even through the acquisition of genetic mutations conferring different levels of protection against the parasite. These include heterozygous carriers of sickle hemoglobin, α- and β-thalassemia, G6PD deficiency and mutations to complement receptor 1 [22]. During our hard-fought battle with malaria, more than 1200 different species of medicinal plants have been employed to treat what was described historically as cyclic fevers and what we can now positively identify as malaria [1]. 

Herbal medicines were the staple malaria treatment predating the identification and purification of the first active antimalarial compound in 1820 [23]. Despite the advancements in medicinal chemistry, compounds found in modern malaria treatments developed in the past century still continue to be based predominantly on derivatives extracted from the same plants that were once used to treat malaria and fevers [24]. Quinine, largely used as a malaria prophylaxis by Europeans in the 1850s, was first identified from the bark of the Cinchona tree, local to Central and South America, alongside other alkaloids such as quinidine, cinchonine and cinchonidine [25]. Extracts from this bark had long been used to calm fevers experienced by indigenous peoples in these locations. Inspired by this use, scientists attempted to extract compounds from the bark. The first attempts at extracting quinine were not overly successful and instead lead to the production of the dye methylene blue and thus fueling the rise of the dye industry [26]. While not directly aiding in the combat against malaria, the development of this dye led to advancement in the field of microbiology, allowing for the coloration of microorganisms and facilitating their observation by microscopy [27]. The successful extraction of quinine proved to be only a starting point for the development of antimalarials that have served as front-line treatment for malaria over the last century. Quinine actually served as a lead structure for the development of chloroquine, mefloquine, pyrimethamine, proguanil, atovaquone and primaquine, by donating its quinoline alkaloid group [1]. The apparition of resistance to a large number of these drugs, most prominently chloroquine, led to a push to develop better antimalarials. Taking inspiration from ancient Chinese medical texts, artemisinin was isolated from the leaves of the sweet wormwood *Artemisia annua* in 1971. This breakthrough proved to clear blood stage parasites with an efficiency that had never been observed before [28]. Artemisinin is a compelling compound and possesses an unusual endoperoxide group that has since been exploited for the synthesis of longer-acting molecules, leading to the development of artesunate [29]. 

Today, artemisinin-based combination therapies (ACTs) are the front-line treatment for *P. falciparum* malaria. In this line of treatment, artemisinin is combined with a second drug that employs a different mechanism of action [30,31,32]. Combination therapies are used as a direct response to the rapid emergence of resistant parasites that appear when using monotherapies as previously seen with chloroquine and pyrimethamine throughout Africa [33]. This treatment remains safe and effective in most endemic regions and has a cost as low as USD 0.30 per child allowing accessible treatment [34]. However, because of widespread use, resistance to these drugs has been emerging in different regions such as South-East Asia and more recently resistant parasites have emerged independently in Africa [35,36]. Problems with accessibility are becoming more common due to the cost and availability of drugs resulting in a rise in counterfeit medications. As a direct consequence, a renewed interest in self-medicating in heavily affected regions is growing. Most commonly employed are infusions of *Artemisia annua* in Asia and *Artemisia afra* in Africa [37]. Self-medicating with natural products can be risky as plants can produce unexpected secondary metabolites that can be dangerous, highlighting the necessity to exploit these products and identify metabolites that are strictly active against the parasite and not the host [34]. Narratives of disease treatment and prevention by these crafted treatments have occasionally pushed researchers to look further into the phytochemicals present in such infusions. What they found surprisingly was substantially more than just artemisinin: terpenes, flavonoids, phenolic acid esters and coumarins were all present in high concentrations and could be interesting compound leads [38]. 

Due to the increase in antimalarial resistance, the accelerating spread of disease and the lack of a commercialized vaccine, new malaria treatments are needed now more than ever before. Natural products sourced from tropical regions have proven themselves an excellent starting point for the development of novel antiparasitics. Recent advancements have accelerated the ability to identify potential lead compounds, unlocking new possibilities for novel drug development. The parasite’s genome has been fully sequenced, unveiling information that was previously unknown helping in identifying potential drug targets [39]. A certain level of automation of assays conducted on live parasites has also been developed, allowing for rapidity in screening new molecules and determining which could potentially lead to clinical candidates [40,41]. DNA barcoding has also gained popularity and the WHO actually recommends applying DNA barcoding, where short characteristic DNA sequences, called barcodes, allow for the identification of species [42], to guarantee the quality of medicinal plants and identify new potential compounds. This technology has evolved during the past decade, starting from single genes to genomes and now metabarcoding [43]. 

The past underlines the fact that for the most part, the development of antimalarials has focused on the identification of natural product scaffolds, which have then been exploited to create efficient new treatments. Indeed, more than 50% of the synthetic drugs on the market are based on products initially derived from plants [44]. Unfortunately, screening natural products at random does not typically provide a good starting point as most extracts have low ligand efficiency and low potency [45]. But the analysis of natural products still has many advantages. The natural world harbors an important library of potential new pharmacophores. The discovery of molecules such as these are immensely important in the battle against the parasite. Another advantage that cannot be overlooked is the acceptance of natural products by large populations of people as they are inherently deemed as being safe as they have been used in disease endemic countries for many generations. This means that anecdotal evidence supports their use within the community. Many communities exhibit a strong desire to share their experience and knowledge to develop new treatments. Their inclusion is important when moving forward in scientific research [46]. There are still unexploited and unexplored environments that could prove very useful in the discovery of antiparasitic drugs.

## 3. Extreme Environments and Their Potential to Fight Disease

Naturally, most plants that have been exploited for the development of antimalarials were found in endemic regions, usually tropical or sub-tropical, as these plants were previously used by the populations residing there. However, investigating new areas may provide novel insights into drug development by bringing previously unexplored potential antiparasitic molecules into focus. 

Extreme environments are defined by their unique environmental conditions that make sustaining any form of life difficult. These extreme environments usually harbor extreme cold or hot temperatures, high pressure, high levels of salinity and difficult access to nutrients essential for life [47]. Organisms that are able to thrive in these environments are pushed to develop strategies to cope with such harsh conditions, often resulting in the production of interesting bioactive molecules. The search for new bioactive molecules from extreme biospheres remains very challenging for several reasons, ranging from field exploration to the cellular and molecular demands that the organisms living in these regions require for their culture and study. In the past decade, advances in the field of extremophile research allowed for the development of exploration techniques, sampling and isolation strategies needed to study these organisms. The study of aerosolized particles led to a better understanding of the climate conditions of these regions as well as the microbial ecosystems present [48,49]. Deep-sea cameras equipped with the capability to detect high-pressure environments [50] and hot-water ice drills have allowed for the retrieval of sub-ice seabed samples [51] and have enabled the exploration of aquatic environments. Further characterization of extremophile organisms was made possible thanks to the advances in ‘omics’ [52]. 

The Arctic and Antarctic regions correspond to 14% of the total biosphere and maintain average temperatures of below 0 °C [53]. It was once believed that life was not sustainable in these polar deserts due to the effects of the extreme cold; however, exploring these regions revealed that some organisms can survive in temperatures as frigid as −20 °C [54]. These polar regions were considered to be plateaus of unchanging terrain devoid of most forms of life. Upon further investigation a large variety of geological variation is observed in the type of sediment, mix of ice and snow, degrees of salinity, different levels of nutrient availability and thermal values, making life in fact possible [55]. Diverse microbial ecosystems adapted to these environments were discovered. Between 2001 and 2004, the Armi Project led by the Finnish Forest Research Institute (Metla) allowed for the isolation of more than 500 different microbial strains from the Arctic region [56]. These organisms are able to thrive under difficult conditions through adaptations they have acquired, whether it be metabolic or structural, modulating the composition of their membrane and favoring specific amino acids during protein synthesis [56]. 

Cryophilic microorganisms have to adapt their molecular and structural biology to overcome challenges due to the extreme cold environment. Challenges encountered by these organisms can include reduced enzyme activity, decreased membrane fluidity and protein denaturation [54]. The main adaptation seen is an increase in flexibility both in individual proteins and protein complexes. These microorganisms tend towards exhibiting proteins and protein structures with lower hydrophobicity and weaker inter-domain and inter-subunit interactions at the core of the protein, while an increase in hydrophobicity in the outer surface of the structure is often present to compensate for the general decrease in stability due to weak interactions [47]. Amino acid preference is also swayed to increase flexibility as shorter neutral chain amino acids are favored. Proteins are often composed of higher amounts of proline and glycine residues and lower amounts of cysteine, lysine and arginine residues, as these residues increase the rigidity of secondary structures [47,56]. This is an important concept for the function of enzymes. Characteristically, enzymes have temperature-specific functions mostly linked to their flexibility and different temperatures can decrease the biocatalytic reaction rates. The higher flexibility seen can fix this problem, allowing for the appropriate function of enzymes at extreme temperatures. Microorganisms living in these extreme temperatures also modify the fluidity of their membranes by an increase in mono- or poly-unsaturated fatty acids [54]. Di Lorenzo et al. reported uncommon structural features of a lipopolysaccharide found in the outer membrane of Gram-negative bacteria called lipid A in three different extremophile bacteria. As lipid A is known to cause an immune response in mammalians, it represents a natural immunomodulatory molecule that could be an interesting scaffold for drug synthesis, as presented in Table 1 [57]. 

The adaptations will also lead to secondary metabolite production for many of these organisms. Secondary metabolites are products of low molecular mass produced by the secondary metabolism often triggered by nutrient-limiting conditions, environmental stress and difficult environmental conditions. While often not considered essential for growth, these molecules exert a specific function ensuring survival in difficult conditions [66]. The biological characteristics of the secondary metabolites produced are interesting and could be used for drug development as these are often toxins, antibacterial agents, serve in the transport of metals, have anticancer properties or have immunomodulating or immunosuppressant properties [67]. Indeed, some secondary metabolites from Psychrophiles, microorganisms able to have optimal growth at extremely cold temperatures, have already been shown to have an antimicrobial effect against some pathogens [68]. Pigments isolated from bacteria found in Antarctic lakes have also proven to be interesting leads in the development of tuberculosis treatments thanks to their antimycobacterial properties. Two such pigments are violacein, isolated from *Janthinobacterium* sp. Ant5-2 (J-PVP), and Flexirubin, isolated from *Flavobacterium* sp. Ant342 (F-YOP) (Table 1) [58]. Another example is diketopiperazines from *Oidiodendon tuncatum*, a fungus from Antarctica with cytotoxic, immunomodulatory, antiviral, antimicrobial and antiproliferative activities (Table 1) [59]. 

Alongside microorganisms, plants also produce very diverse metabolites, which depend largely on their environment, the weather conditions and the season of harvest. These compounds are either non-volatile or volatile. Plants able to grow in extreme environments display characteristics that others do not and demonstrate interesting properties as antioxidants, anti-inflammatories and antimicrobials [69]. Hopefully, antiparasitic properties will also be identified upon further investigation. The most diverse secondary metabolites produced are terpenoids, phenols and alkaloids. Terpenoids, more specifically those containing lactones, are particularly interesting because these types of compounds are already being used as antimicrobials, in cancer treatments and notably found in the antimalarial drug artemisinin [70]. Phenols have multiple uses as well. They form the precursor of aspirin, have an antiviral effect on HIV-1 and can be used during cancer treatment [71].

## 4. The Boreal Forest: An Unexplored Potential Treasure Trove of Natural Remedies 

Despite extensive research into tropical plants and those found at the extreme poles of the Earth, the secrets of the Canadian boreal forest remain largely unknown. 

The boreal forest, comprising 60% of Canada’s landmass, stands as one of the largest biomes globally and remains one of the most extensive intact forest and wetland ecosystems on Earth, making it a haven for a rich diversity of flora and fauna [72]. Dominated primarily by coniferous species such spruce, pine and fir, it is also home to a variety of mosses, lichens and shrubs, contributing to its botanical richness. Characterized by severe winters with prolonged snow cover and short warm summers, the region experiences extreme temperature fluctuations [73], with a significant portion of land covered by permafrost [74]. The environmental conditions of the boreal forest are shaped by its weather patterns and have led to the evolution of ecosystems adapted to extreme cold and limited sunlight. As previously mentioned, species within this type of harsh habitat often exhibit adaptations to survive challenging environments, producing unique chemical compounds like alkaloids and terpenoids. These molecules present in plants from the boreal forest represent potentially attractive targets in the search for novel treatments for a wide range of diseases [21]. 

These interesting properties are not new and have been used by indigenous peoples inhabiting the boreal forest in traditional healthcare systems without knowing the active ingredients present. In fact, about 2500 plants from different regions of the boreal forest are known to have been used for their different medicinal effects [75,76]. Different remedies were prepared from different plant parts, with almost all plant parts dedicated to a specific treatment, whether it be the whole plant, roots, rhizomes, bark, leaves or the fruit of the plant. The roots of the plants were employed the most often for the production of remedies [21]. 

Recently, a renewed interest in the exploitation of natural molecules from the boreal forest has resurfaced due to the resurgence of diseases such as tuberculosis. Going back to familiar grounds seems appropriate to many [77]. During a study, a total of 96 endophytes were isolated from the leaves of 12 Canadian medicinal plants and showed an inhibitory effect of *Mycobacterium tuberculosis* growth [77], showing promise for the exploitation of boreal forest molecules for drug development. Additionally, in the literature review published by Uprety et al. in 2012, the authors were able to identify 546 medicinal plant taxa used by indigenous peoples in the Canadian boreal forest. The latter were active against 28 diseases and disorders, mostly gastrointestinal in nature [21]. Despite the large diversity of plant species used throughout the Canadian boreal forest, certain of these plants are commonly used by a number of different communities across the country (Table 2).

A portion of these medicinal plants have been studied extensively by researchers. One context of the utilization of molecules from the boreal forest by indigenous peoples involves the use of medicinal plants with antioxidant activity to treat symptoms of diabetes and its complications. The antioxidant activity of these traditional medicinal plants may stem in part from antioxidant vitamins, phenolics, or tannins. In a study conducted by McCune and Johns in 2002, 35 species of plants traditionally used by Indigenous communities were selected, revealing that 89% of the methanolic extracts of these plants exhibited significantly higher activity than common modern dietary components, with 14% statistically equivalent to ascorbic acid and 23% demonstrating activities similar to green tea and a positive control for vitamin E, Trolox [79], proving this an actual viable treatment for pre-diabetes, as was thought by the indigenous communities. Another species that has been extensively studied is *Rhododendron tomentosum* spp. *Subarcticum*, commonly known as Northern Labrador tea. This plant species is of particular interest because it is still widely used today. It exhibits antioxidant and anti-inflammatory properties which have been proven helpful for the treatment of type II diabetes, various respiratory illnesses, different infections, to relieve stomach and tooth aches and the general relief of cold symptoms (Table 2) [76,78], depending on which part of the plant is used. In addition to plants, medicinal molecules from the boreal forest can originate from other organisms. More recently, the potential of *Haploporus odorus* (*Agaricomycetes*), a lesser-known polypore, has been discovered. This fungus can be found in the taiga flood lands and broadleaf forests of the Northern Hemisphere and produces haploporic acid A, a substance that can be used in cancer therapy (Table 1) [60]. 

## 5. The Paradigm of Using Molecules Extracted from Northern Canada to Treat Malaria

It is not a unique concept to use plant-derived molecules to treat parasitic diseases. As mentioned previously, quinine was once widely used to treat malaria and artemisinin-based combination therapy is still the front-line treatment. Both of these molecules were originally derived from plants. As seen with both of these treatments, inspiration is mostly drawn from tropical or sub-tropical regions. With no commercialized vaccine against malaria and the apparition of resistant parasites to front-line treatments, it is necessary to find new treatment options and therapeutic alternatives. 

Despite the growing interest in the development of novel treatments inspired from northern plants and those found in the boreal forest to treat an array of infectious diseases, much less is known in regard to the antiparasitic effects that these molecules could have. But in this lies an advantage. These polar organisms are also seldom used against tropical diseases such as malaria, reducing the probability of pre-existing resistance and similarity to current drugs. Therefore, molecules found in northern environments represent an opportunity and a potentially viable option for the development of antiparasitic treatments. 

When conducting research on the database PubMed using keywords such as ‘boreal’, ‘forest’, ‘Canada’, ‘Canadian’, ‘North’, ‘antiparasitic’, ‘antiplasmodial’ and ‘molecules’, an increased interest in these topics is apparent in the past decade. This shows a change in how researchers are striving to find new antimalarials. While many of these studies concentrate on anti-inflammatory, antioxidant, antibacterial and antiviral properties, a few do describe antiparasitic activities that could be exploited to combat parasitic diseases, including malaria. 

As mentioned previously, Labrador tea, *Rhododendron tomentosum* spp. *Subarcticum*, is widely used and displays many interesting properties. The essential oil or its isolated compounds have not been extensively studied yet with regards to antiparasitic activity. However, during preliminary studies, it has proven to display some antiplasmodial activity, as described in Table 3 [80]. Growth inhibition assays against different strains of the malaria parasite *P. falciparum*, including the chloroquine resistant strain Dd2, were conducted using either *R. subarticum*’s essential oil or the isolated compound ascaridole. In this case, it was confirmed that the essential oil’s activity was attributed to the ascaridole content [80]. This finding is not surprising as several essential oils have already been proven to be efficient antiparasitics, having effect against leishmaniasis [81], amoebiasis [82], Chagas’ disease [83] or malaria [84]. Also, extracts from *Stereocaulon paschale*, a lichen found in Nunavik, Canada, showed some antimicrobial activity against oral pathogens such as *Porphyromonas gingivalis* and *Streptococcus mutans* (Table 1) [61]. While not tested against parasites, the unique lichen metabolites isolated could prove interesting in the development of new antimalarials.

Appreciably more research has been conducted on the identification and characterization of molecules from organisms in marine polar environments. These compounds from the natural world are active against different parasites and could serve as inspiration for the development of antimalarials. Recently, mortiamides isolated from *Mortierella* species found in marine sediments from Northern Canada have been tested and have shown promising antiplasmodial activity (Table 3) [85,86]. Alongside this, clindamycin, a structural analogue of mortiamides, has also been investigated [87]. Compounds from the Antarctic deep-sea octocoral *Cnidaria*, like keikipukaldes, pukalide aldehyde and norditerpenoid ineleganolide, have been proven to be active against *Leishmania donovani* (Table 1) [62]. Spongian diterpenoids from the Antarctic sponge *Dendrilla antarctica* have also been found to be active against *Leishmania donovani* at micromolar concentrations (Table 1) [63,88]. Norselic acids A–E found in an Antarctic sponge (Table 1) [64] were also active against the *Leishmania* parasite in low micromolar activity [20]. Tricyclic sesquiterpenoids, shagenes A and B, found in a yet undescribed Antarctic soft coral have been found to exhibit some antiparasitic activity (Table 1) [20,65]. These examples could provide deeper insight into the development of novel antimalarials.

## 6. Conclusions

Historically, humans have used natural products to fight disease and alleviate their symptoms. Diverse preparations were made with plants, such as infusions, decoctions and essential oil extraction depending on what symptoms were being treated. The preparation type and components change slightly when treating gastro-intestinal disorders, musculoskeletal disorders, colds, sore throats, injuries or infections [21,89]. 

Until recently, tropical and sub-tropical environments were mostly used for inspiration when developing therapeutic treatments in the pharmaceutical industry. This is primarily explained by the widespread distribution of diseases present in these regions and the remedies commonly used by the indigenous peoples. In fact, most of the current drugs were at first inspired from bioactive plant compounds, for example, the well-known antimalarial, Artemisinin. Meanwhile, northern environments have remained largely unexplored and unexploited, leaving a breach in potential discoveries and leads.

The increase in antimalarial resistance and the lack of a commercialized vaccine makes the discovery of new treatments urgent. The exploration of the extreme cold environments encountered in Northern Canada offers new opportunities for the discovery of next-generation antiparasitics. As previously mentioned, extreme environments harbor interesting reservoirs of organisms with exploitable attractive properties for use in the biotechnological, pharmaceutical, cosmetic and bioremediation sectors [90]. During the past decade, further investigation of choice northern environments has unearthed promising bioactive compounds. For example, two effective molecules against *P. falciparum*, the essential oil of *Rhododendron tomentosum* spp. *Subarcticum* from Nunavik [80] and mortiamides isolated from *Mortierella* found in Nunavut [87], have been recently brought to light.

## 7. Challenges

Despite the alluring properties demonstrated by many molecules extracted from Northern Canada, many challenges remain in their exploitation. These obstacles range from the exploration of the unfriendly terrain found in these environments to the laboratory cultivation and study of unruly extremophile organisms, and this in spite of the many advancements that have been made in the past decade. Northern Canada has characteristically rough and vastly uninhabited terrain, making it difficult to explore safely. Extremophile organisms often have particular and mostly unknown metabolic needs that are difficult to recreate in a laboratory setting, limiting their characterization to only what can be observed in the field. Some improvement has been made. Sampling techniques and isolation methods are being developed alongside different ‘omics’ strategies that allow for the discovery and characterization of an increasing number of organisms [91]. These organisms often display interesting antioxidant, anti-inflammatory, antibacterial and antiparasitic properties, as previously demonstrated, and could be further exploited as a potential source of novel antimalarials. This is important as the push for the discovery of new classes of antimalarials with unique mechanisms of action is increasing. Recycling old compounds is becoming less and less feasible due to the increase in resistance observed in *Plasmodium* parasites; it thus would be of interest to further the characterization of certain of the molecules present in this review and evaluate their efficacy against *P. falciparum*, as was conducted with the essential oil and isolated compounds from *Rhododendron tomentosum* spp. *Subarcticum* and *Mortierella*. 

In addition, the scientific community needs to take care when exploiting this resource with the environment and the indigenous people inhabiting it in mind. Indigenous communities continue to rely on traditional medicines. In general, there has been an important lack of acknowledgement of Indigenous traditional knowledge during scientific research studies [21,92,93]. Whether it be for worries of cultural appropriation or the absence of the sharing of profits arising from commercialization despite their involvement, indigenous peoples are often hesitant when approached by scientists [94]. Research teams have shown interest in learning new approaches. However, these challenge certain beliefs held by the indigenous communities. It would be important to forge links within the communities and rebuild trust that has often previously been broken. The boreal forest has been respectfully exploited for thousands of years by indigenous peoples as it comprises an impressive diversity spread over the vast territory it occupies [21], and this must not change. 

## Figures and Tables

**Table 1:** Polar organisms producing bioactive molecules and their use in the treatment of different ailments and infections.

Species/Location	Molecule	Properties	Ref
Gram-negative bacteria Ex. *Psychrobacter cryohalolentis* *Pseudoalteromonas * Bacteria found in Antarctica	Lipid A Lipopolysaccharide	Immunomodulatory molecule Immunostimulatory (TLR4/ MD2 pathway) Activates TNF production	[57]
*Janthinobacterium* sp. Ant5-2 J-PVP Bacteria isolated from Antarctic lakes	Violacein Pigment	Antimycobacterial (ex. Tuberculosis) *Mycobacterium tuberculosis* mc26230 Minimal inhibitory concentration (MIC) 5 μg/mL *M. tuberculosis* H37Rv MIC 34.4 μg/mL	[58]
*Flavobacterium* sp. Ant342 F-YOP Bacteria isolated from Antarctic lakes	Flexirubin Pigment	Antimycobacterial (ex. Tuberculosis) *M. tuberculosis* H37Rv MIC 10.8 μg/mL	[58]
*Oidiodendon tuncatum* (GW3-13) Fungus isolated from Antarctic soil	Epipolythiodioxopiperazines Diketopiperazines	Cytotoxic Immunomodulatory Antiviral Antimicrobial Antiproliferative Cytotoxic activity against 5 cancer cell lines HCT-8, Bel-7402, BCG-823, A-549, A-2780 Various IC50 ranging from 0.003 μg to 1.83 μg/mL Cytotoxic effect depending on the presence of a sulfide bridge within the molecule	[59]
*Haploporus odorus * Agaricomycetes Northern Hemisphere	Haploporic acid A	Cancer therapy Proliferation pathways are affected coordinating the arrest of the cell cycle often resulting in the apoptosis of cancerous cells	[60]
*Stereoculon paschale* Lichen Northern Canada	Pseudodepsidone-type metabolites Lobaric acid	Antimicrobial activity against selected oral pathogens *Porphyromonas gingivalis* *Sterptococcus mutans* MIC ranging from 20 to 80 μM	[61]
*Plumarella delicatissima* Cnidaria Antarctica	Keikipukaldes Pukalide Aldehyde Norditerpenoid ineleganolide	Antiparasitic activity against *Leishmania donovani* IC50 against *L. donovani* 1.9 to 12 μM	[62]
*Dendrilla antarctica* Sea sponge Antarctica	Diterpenoids Tetrahydroaplysulphurin-1 Membranoids B, D, G	Antiparasitic activity against *Leishmania donovani* Tetrahydroaplysulphurin-1 IC50 3.5 μM Membranoid B IC50 0.8 μM Membranoid D IC50 1.4 μM Membranoid G IC50 1.9 μM	[63]
*Crella* sp. Sea sponge Antarctica	Norselic acid A–E	Antiparasitic activity against *Leishmania donovani* Norselic acid A IC50 2.5 μM Norselic acid B IC50 2.4 μM Norselic acid C IC50 2.6 μM Norselic acid D IC50 2.0 μM Norselic acid E IC50 3.6 μM	[64]
Undescribed Antarctic soft coral Scotia Arc, Antarctica	Tricyclic sesquiterpenoid Shagene A	Antiparasitic activity against *Leishmania donovani* IC50 5 μM	[65]

Abbreviations: TLR4, toll-like receptor 4; MD2, myeloid differentiation factor 2; TNF, tumor necrosis factor; MIC, minimal inhibitory concentration; IC50, half-maximal inhibitory concentration.

**Table 2:** Most commonly used traditional and medicinal plants by the Aboriginal people inhabiting the Canadian boreal forest [21].

Species/Common Name	Parts Used	Preparations	Primary Uses
*Abies balsamea* Balsam fir	Gum Sap Branches Needles Cones Bark Roots Buds	Salve/Ointment Poultice Infusion Dried/Powder Decoction	Application as topical treatments for sores and cuts Arthritis Muscular pain Stomachache, nausea and colic Tuberculosis
*Achillea millefolium* Yarrow	Whole plant Leaves Roots Flowers	Salve/Ointment Dried/Powder Decoction Infusion Poultice Burned/Smoked	Fever Respiratory illnesses Aches and pains Arthritis Migraines Treatments for sores and cuts
*Acorus calamus* Sweet flag	Roots Rhizome	Scalded Infusion Tonic Dried/Powder Decoction Smoked	Cold and flu symptoms Fever Inflammation Topical treatment for sores, cuts and infections Aches and pains Treatment of parasitic intestinal worms
*Aralia nudicaulis* Wild sarsaparilla	Leaves Roots Stalk Rhizomes	Infusion Tonic Decoction Dried/Powder Poultice	Pneumonia Weakness Aches and pains Cold and flu symptoms Stomachache Topical treatment of wounds, infections and sores
*Betula papyrifera* Paper birch	Leaves Stem/Bark Buds Wood Roots Sap	Decoction Dried/Powder Infusion Poultice Salve/Ointment	Topical treatment for stings, cleanser, rashes and infection Stomachache Tonsilitis Cough
*Cornus sericea* Red-osier dogwood	Bark Roots Stems Fruits/Pith Twigs Leaves	Infusion Decoction Smoked	Diarrhea Topical treatment for poison ivy, sores and stings Sore throat Cold and flu symptoms Fever Weakness Stomachache Tuberculosis
*Heracleum maximum* Cow parsnip	Roots Leaves Flowers	Infusion Dried Decoction Paste Poultice	Cholera Topical treatment for sores, boils and infections Cold and flu symptoms Tooth ache Sore throat Inflammation Smallpox Tuberculosis Headache
*Juniperus communis* Juniper	Fruits Roots Leaves Bark Stem Gum	Infusion Juice (berries) Decoction Dried/Powdered Poultice Tonic Smoked	Tuberculosis Cold and flu symptoms Stomachache Aches and pains Topical treatment for skin problems, boils and wounds Fever
*Larix laricina* Tamarack	Branches Bark Needles Gum Leaves Cones Sap Roots Pulp	Infusion Decoction Poultice Inhalation	Topical treatment of boils, wounds, frostbite, infection and burns Stomachache Cold and flu symptoms Anemia Gonorrhea Inflammation Sore throat Ache and pains Jaundice Arthritis
*Mentha arvensis* Wild mint	Whole plant Flowers Leaves Stem	Infusion Tonic	Stomachache Topical treatment of sores and infection Cold and flu symptoms Weakness Diarrhea Fever Toothache
*Nuphar lutea* Yellow water lily	Rhizomes Roots Whole plant Stems	Dried/Powder Infusion Poultice	Arthritis Inflammation Topical treatments of boils, infection and stings Aches and pains Cold and flu symptoms Stomach pain
*Picea glauca* White spruce	Twigs Bark Sap Gum	Infusion Decoction Poultice Salve/Ointment Dried/Powder	Fever Cold and flu symptoms Headaches Joint pains Topical treatment for sores, burns, irritation, infection and wounds Sore throat Toothache Intestinal problems Aches and pains
*Picea mariana* Black spruce	Twigs Sap Bark Charcoal Cones/Young tips Leaves Roots Gum	Infusion Decoction Salve/Ointment Dried/Powder	Cold and flu symptoms Fever Topical treatment for boils, sores, infections and burns Stomachache Toothache Sore throat
*Populus balsamifera* Balsam poplar	Buds Sap Bark Leaves Roots Catkins Rotten wood	Infusion Salve/Ointment Decoction Poultice	Internal blood diseases Topical treatment for frost bite, sores, infection, skin diseases and stings Toothache Aches and pains Seizures Stomachache Inflammation
*Populus tremuloides* Quaking aspen	Sap Bark Leaves Buds Seeds Roots Rotten wood	Infusion Decoction Poultice Tonic Dried/Powder	Treatment of intestinal parasitic worms Stomachache Cold and flu symptoms Food poisoning Fever Topical treatment of wounds and stings Toothache
*Rhododendron groenlandicum* Labrador tea	Whole plant Leaves Roots	Infusion Decoction Tonic Dried/Powder Salve/Ointment	Cold and flu symptoms Pneumonia Whooping cough Ache and pains Arthritis Topical treatment of wounds, sores, burns Sore throat
*Salix* sp. Willow	Bark Roots Leaves	Dried/Powder Salve/Ointment Infusion Decoction Poultice	Topical treatment for sores, bruises and stings Toothache Arthritis Cold and flu symptoms Aches and pains Stomachache Dysentery
*Sorbus americana* American mountain ash	Leaves Bark Roots Buds Stem	Infusion Decoction Burned Poultice Paste Tonic	Stomachache and colic Sore throat Cholera Aches and pains Topical treatment for boils Cold and flu symptoms Toothache Arthritis Weakness
*Thuja occidentalis* Arborvitae	Wood Leaves Cones Charcoal Bark Gum	Infusion Burned Decoction Salve/Ointment Dried/Powder	Fumigation against disease Fever Toothache Infection Inflammation Cold and flu symptoms Topical treatment for infections, wounds, burns and paralysis Arthritis Colic Convulsions
*Rhododendron tomentosum* spp. *Subarticum* Northern Labrador tea [76,78]	Whole plant	Infusions	Cold and flu symptoms Toothache Stomachache Cough Tuberculosis Throat aches Aches and pains Headache Eye problems Nasal congestion Wound treatment Arthritis Infections Inflammation Weakness Heart and chest pain

**Table 3:** Organisms form Northern Canada producing bioactive molecules with interesting antimalarial properties.

Species/Location	Molecule	Disease and Properties	Ref
*Rhododendron tomentosum* spp. *Subarticum* Isolated compound from essential oil Northern Canada	Ascaridole	Antiplasmodial (3D7 and Dd2 parasite strains) IC50 against 3D7 147.3 ± 7.3 nM IC50 against Dd2 104.9 ± 11.2 nM	[80]
*Mortierella* Fungus Northern Canada	Mortiamides (A, B, D) Clindamycin (Mortiamide analogue)	Antiplasmodial (3D7 and Dd2 parasite strains) Mortiamide A IC50 against 3D7 7.85 ± 0.97 μM IC50 against Dd2 5.31 ± 0.24 μM Mortiamide B IC50 against 3D7 3.16 ± 0.65 μM IC50 against Dd2 2.10 ± 0.18 μM Mortiamide D IC50 against 3D7 1.31 ± 0.12 μM IC50 against Dd2 0.94 ± 0.07 μM	[85,86]

Abbreviations: IC50, half-maximal inhibitory concentration.
